# Morphology, Mechanical Properties and Shape Memory Effects of Polyamide12/Polyolefin Elastomer Blends Compatibilized by Glycidylisobutyl POSS

**DOI:** 10.3390/ma14010027

**Published:** 2020-12-23

**Authors:** Dong-Hun Lee, Young-Wook Chang, Keon-Soo Jang

**Affiliations:** 1 Department of Materials & Chemical Engineering, Hanyang University, Ansan, Gyeonggi-do 15588, Korea; ldh0320@hanyang.ac.kr; 2 BK21 FOUR ERICA-ACE Center, Hanyang University, Ansan, Gyeonggi-do 15588, Korea; 3 Department of Polymer Engineering, School of Chemical and Materials Engineering, The University of Suwon, Hwaseong, Gyeonggi-do 18323, Korea; ksjang@suwon.ac.kr

**Keywords:** polymer blend, polyamide12, polyolefin elastomer, POSS, compatibilization

## Abstract

Small amounts of glycidylisobutyl polyhedral oligomericsilsesquioxane (G-POSS) (up to 10 phr) were added into a immiscible polyamide12 (PA12)/polyolefin elastomer (POE) blend (70 wt%/30 wt%) by simple melt mixing. The effects of the G-POSS on phase morphology and mechanical properties were investigated by FE-SEM, tensile testing, Izod impact test and dynamic mechanical analysis. FE-SEM analysis revealed that domain size of the dispersed POE phase in matrix PA12 is decreased significantly by adding the G-POSS, indicating a compatibilization effect of the G-POSS for the immiscible PA12/POE blend. The PA12/POE blend compatibilized with POSS showed simultaneous enhancement in mechanical properties including tensile modulus, strength and toughness. Further, thermally triggered shape memory effect was observed in this compatibilized blend.

## 1. Introduction

Polymer blending is an efficient and simple way to develop new polymeric materials possessing desirable physical properties and specific functionalities for various end-use applications. Most of the polymer blends are immiscible and need proper compatibilization in order to obtain proper phase separated morphologies which determine the performance properties. The compatibilization induces finer phase morphology by improving interfacial adhesion between the component polymers in the blend, which promotes their synergistic combination [1,2].

Blends of polyamides (PA) with polyolefins (PO) are important class of polymer blends because of their balanced properties of strength, toughness and moisture resistance. For the compatibizations of the immiscible PA/PO blends, functionalized polyolefins, such as maleic anhydride (MA)-grafted polyolefins, have been investigated [3,4,5,6,7,8]. Recently, it has been reported that phase morphology and properties of the immiscible PA/PO blends can be influenced by the addition of inorganic nanoparticles such as clay [9], silica [10], carbon nanotube [11], alumina [12] and POSS [13]. The nanoparticles are located at the interface between the component polymers or in one component selectively, which affect the phase morphology of the blends.

Among various inorganic nanofillers, polyhedral oligomeric silsesquioxane (POSS) is unique which features a well-defined nanosized Si–O cage structure (Si_8_O_12_) with additional organic functional groups covalently bonded to each vertex Si on the cage. Due to the organic functional groups, the POSS cage is naturally compatible with polymers, and can be chemically bonded to polymer chain [14,15,16]. The POSS can be added into various thermoplastics such as polyolefins [17,18,19], polyamides [20,21,22] and others [23,24,25] by melt blending using conventional high-shear mixer and induced improved thermal stability and mechanical properties of the matrix polymer. As compared to the POSS nanocomposites with a single polymer matrix, relatively less studies on those with immiscible polymer blends have been reported [13,26,27].

Polyamide12 (PA12) is one of the important engineering plastics with a broad range of applications including oil resistant tubes for automotive, cables, food packaging films and powder coatings for metals. For the improvement in toughness of the PA12, blending it with various rubbers such as styrene/ethylene-butylene/styrene block (SEBS) rubber [28], epoxidized natural rubber (ENR) [29] and natural rubber [30] has been explored However, this decreases the tensile modulus and strength. Polyolefin elastomer (POE), a commercially important rubber developed by Dow Elastomers under the brand name Engage^®^, has good processibility and thermo-oxidative resistance. To the best of our knowledge, studies on PA12/POE blend with simultaneously improved toughness and strength, which endow the material to have advantages in practical applications, have not been reported yet.

**Figure 1:**
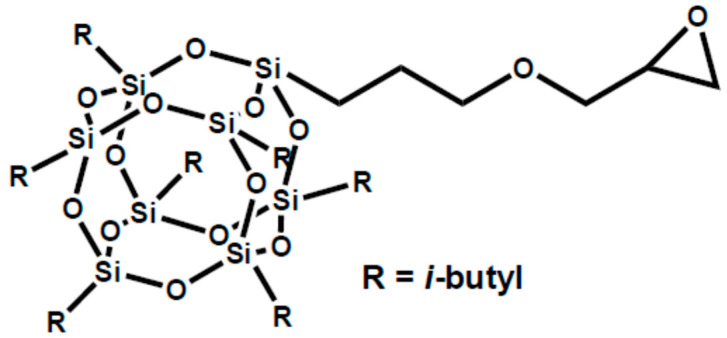
Chemical structure of glycidylisobutyl polyhedral oligomericsilsesquioxane (G-POSS).

Hence, this work employed glycidylisobutyl POSS (G-POSS) having a cage structure with one glycidyl and seven isobutyl groups at each corner of the cage (Figure 1) as a compatibilizer and reinforcing nanofiller for immiscible PA12/POE blend. The G-POSS was expected to act as a compatibilizer for the blend due to its affinity with both component polymers via reaction of its glycidyl group with end reactive groups (–NH_2_ or –COOH) of PA12 during melt mixing and van der Waals interaction of isobutyl groups with POE, respectively. The compatibilization and reinforcing effects of the G-POSS for the PA12/POE (70 wt%/30 wt%) blend were investigated by observation of phase morphology and mechanical properties. A thermally-triggered shape memory effect of this blend is also reported.

## 2. Materials and Methods

### 2.1. Materials and Sample Preparation

PA12 (Rilsan AESNO TL) was purchased from Arkema (Paris, France). POE (Engage 8842, Dow Chemical Co., Midland, MI, USA) was obtained from Dow Chemical Co., USA. Glycidylisobutyl POSS (G-POSS, EP0418) was purchased from Hybrid Plastics Co (Hattiesburg, MS, USA).

PA12/POE (70 wt%/30 wt%) blends with G-POSS content of up to 10 part per hundred (phr) of the polymers were prepared by melt mixing at 200 °C in a Haake internal mixer equipped with a cam rotor (Haake Polylab Rheomix 600, Karlsruhe, Germany) at a rotor speed of 60 rpm. PA12 was first melted for 1 min followed by addition of POE and mixed for another 1 min, then desired amount of G-POSS was loaded and mixed into the mixture and mixing continued for 10 min till the mixing torque was stabilized. The obtained PA12/POE/G-POSS mixture was formed into a sheet by compression molding at 200 °C in an electrically heated press (Carver 2518, Wabash, IN, USA) for the property measurements.

### 2.2. Characterization

#### 2.2.1. FE-SEM Analysis

Phase morphologies of the blend were investigated by a field emission-scanning electron microscope (FE-SEM, S-900, Hitachi Co., Tokyo, Japan) at an accelerating voltage of 15 kV. To avoid ductile deformation during fracture, the samples were chilled in liquid nitrogen before breaking to initiate a brittle fracture. The cryogenically fractured surface was sputter-coated with platinum prior to SEM observation.

#### 2.2.2. Tensile Test

Tensile properties were measured at 25 °C using a universal testing machine (STM-10E, United Co., Fullerton, CA, USA) at a crosshead speed of 50 mm/min with an initial gauge length of 20 mm. At least five specimens were used for the test. Elastic moduli were obtained from the initial slope of the stress–strain curves (up to about 1% strain). Tensile toughness of each sample was obtained by calculating the area of the stress–strain curves.

#### 2.2.3. Izod Impact Test

Notched Izod impact strength was measured using a sample with a thickness of 4 mm and width of 10 mm at 25 °C using an impact tester (DYC-103C, Daeyeong MTC, Hwaseong, South Korea) according to ISO 180. The test was conducted using 10 specimens, and the average values are presented here.

#### 2.2.4. Dynamic Mechanical Analysis

Dynamic storage modulus and tan δ as a function of temperature were determined using a dynamic mechanical analyzer (DMA2980, TA Instruments, New Castle, DE, USA) under a cyclic tensile strain with an amplitude of 10% at a frequency of 1 Hz. The temperature increased at a heating rate of 2 °C min^−1^ from −100 to 200 °C.

#### 2.2.5. Evaluation of Thermally-Triggered Shape Memory Effects

In order to evaluate thermally-triggered shape memory behavior of the samples, temporarily-fixed sample obtained by uniaxial deformation of dogbone-shaped specimen with a thickness of ca. 1 mm to 50% at 70 °C (which is just above *T*_g_ and well below *T*_m_ of the blend) followed by cooling the deformed shape to 0 °C was heated to 70 °C. The shape fixing ratio (*R*_f_) and shape recovery ratio (*R*_r_) of samples were determined by following equations, respectively.

Shape fixing (%) = ε_u_/ε_m_ × 100 (1)

Shape recovery (%) = [(ε_m_ − ε_p_)/ε_m_] × 100 (2)

where ε_m_ is an initial strain imposed onto the sample (50% in this case), ε_u_ is a strain measured upon cooling the deformed sample at 0 °C and ε_p_ is a strain measured upon heating the temporarily deformed shape at 70 °C.

## 3. Results and Discussions

### 3.1. Phase Morphology

**Figure 2:**
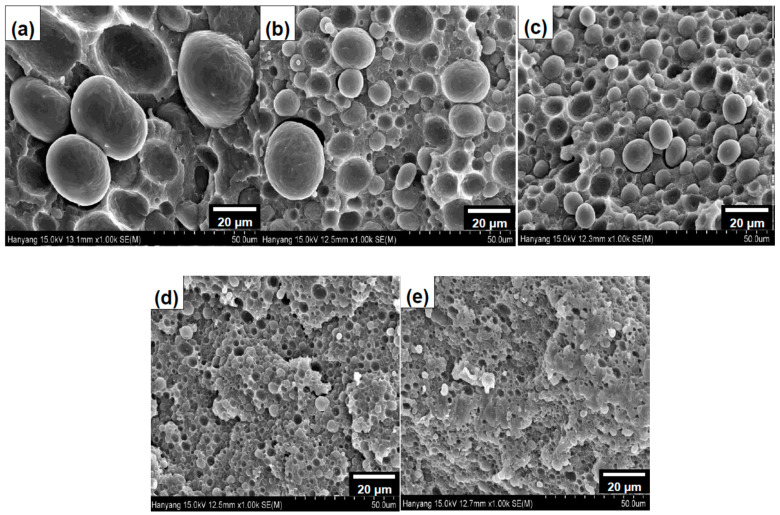
FE-SEM images of polyamide12 (PA12)/polyolefin elastomer (POE)/G-POSS blends with different G-POSS contents of (**a**) 0, (**b**) 1, (**c**) 3, (**d**) 5 and (**e**) 10 phr.

**Figure 3:**
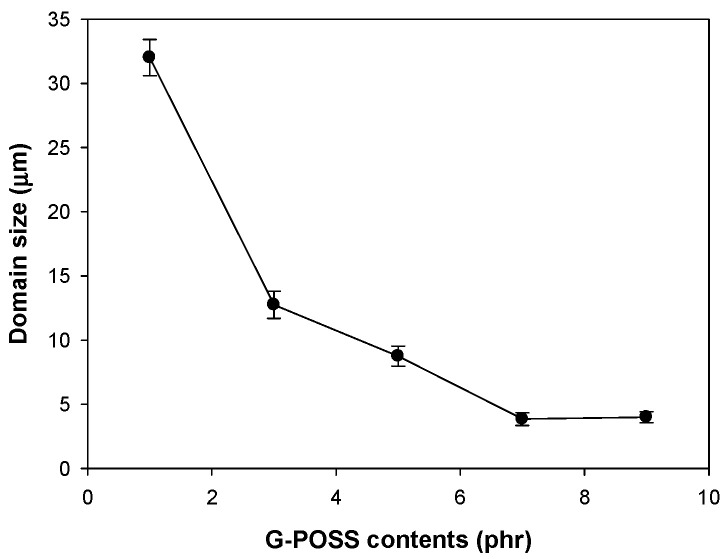
Variation of dispersed domain size in blend with G-POSS content.

SEM images of the blend with various amounts of POSS are shown in Figure 2. It can be seen that all blends have phase-separated morphologies, in which POE forms dispersed domain in PA12 matrix, and the dispersed domain size decreased with addition of a small amount of POSS. The dispersed domain size of POE phase with the amount of POSS are shown in Figure 3. It can be seen that the domain size decreased from 34 μm for unfilled blend to about 10 μm for the blend with 5 phr POSS, and then a slow but gradual decrease was observed with further increasing amount of the POSS. Similar trend was also observed in immiscible blend compatibilized with a block (or graft) copolymer [3] which are located at the interface between the component polymers. The G-POSS nanoparticles might have been located at the interface between PA12 and POE through reaction between glycidyl group of G-POSS with amine end group of PA12 during high shear mixing [13] and van der Waals interactions between alkyl group of POSS with POE [17]. This can prevent the coalescence of the dispersed rubber phase, thereby decreasing the rubber diameter. At G-POSS of higher than 5 phr, the competition between break-up and coalescence was maintained at the same level.

### 3.2. Mechanical Properties

**Figure 4:**
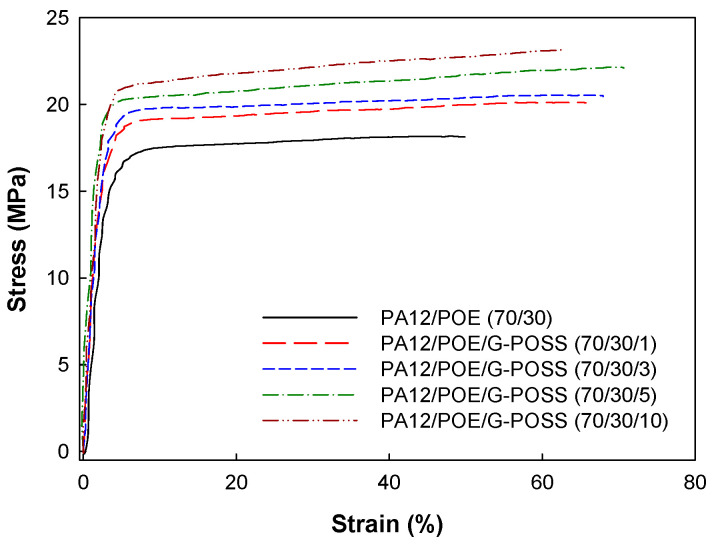
Stress–strain curves of samples.

Stress–strain curves of PA12/POE blend with various amounts of G-POSS are presented in Figure 4, and Young’s modulus, tensile strength, elongation-at-break and tensile toughness obtained from the curves are summarized in Table 1. The blend compatibilized by G-POSS reveals higher values in the modulus, tensile strength and elongation at break, as well as tensile toughness as compared to the blend without the POSS. The Izod impact strengths of the blend are also shown in the Table 1. It can also be seen that the Izod impact strength improved upon the loading of G-POSS into the blend. Tensile modulus, tensile toughness and Izod impact strength of the blend with 10 phr POSS increased by about 115%, 60% and 20% as compared to those of the blend without the POSS, respectively.

Such improved toughness of the blends compatibilized by the POSS is attributed to decrease in dispersed rubber particle sizes as observed in the SEM image as well as enhanced interfacial adhesion due to the G-POSS located at the interface between the component polymers. In addition, the rigid POSS nanoparticles acted as a reinforcing filler for the PA12/POE blends and led to enhancement in strength and modulus. In other words, compatibilizing action of the rigid POSS nanoparticles resulted in outstanding simultaneous enhancement in strength and toughness. Similar simultaneous enhancements in reinforcement and toughness were also observed in other rubber toughened polymer blends containing inorganic nanoparticles [31,32].

### 3.3. Dynamic Mechanical Properties

**Figure 5:**
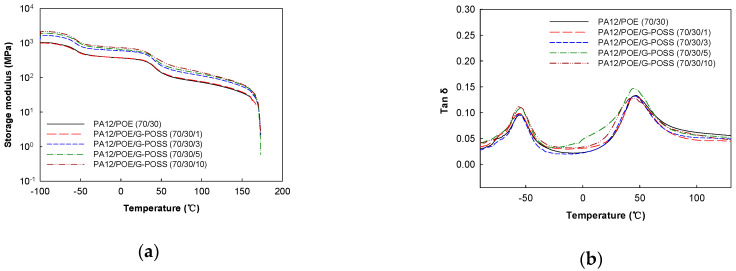
Variation of (**a**) storage modulus; (**b**) tan δ with temperature of samples.

Figure 5a,b show temperature dependence of dynamic storage moduli and tan δ of the samples, respectively. As shown in Figure 5a, the storage moduli of the blend compatibilized by POSS are higher than those of the neat blend over the whole temperature range examined here, and the modulus increased with increasing G-POSS content. The storage modulus at 30 ℃, for example, was enhanced from 296 MPa for unfilled blend to 564 MPa (about 100% increase) for the blend loaded with 10 phr POSS. Such enhancement is correlated with an increase in tensile modulus, which is attributed to finer dispersion of rubber particle due to the compatibilization by the G-POSS and rigid nature of the POSS nanoparticles in the blend. It is also to be noted that all samples exhibit persistent plateau before the temperature reaches 170 °C at which crystalline domains of PA12 are melted.

Variation of tan δ with temperature is shown in Figure 5b. The two peaks appeared in all samples, which are attributed to glass-to-rubber transition of the POE (lower temperature) and PA12 (higher temperature), respectively. The glass transition temperatures (tan δ peak maximum) of the blend samples are shown in Table 2. It can be seen from the table that *T*_g_ of PA12 phase was shifted towards the lower value with the incorporation of POSS into the blend, from 47.1 °C in the neat PA12/POE blend to 41.8 °C in the blend with POSS content of 10 phr. The lowering of *T*_g_ of PA12 phase is due to interfacial compatibilization between the PA12 with POE by the G-POSS, which resulted in increased amorphous portion of PA12 [3,4]. Lowering of *T*_g_ of POE phase in the presence of POSS was probably due to the plasticization effect of isobutyl group of the G-POSS.

### 3.4. Shape Memory Effects

**Figure 6:**
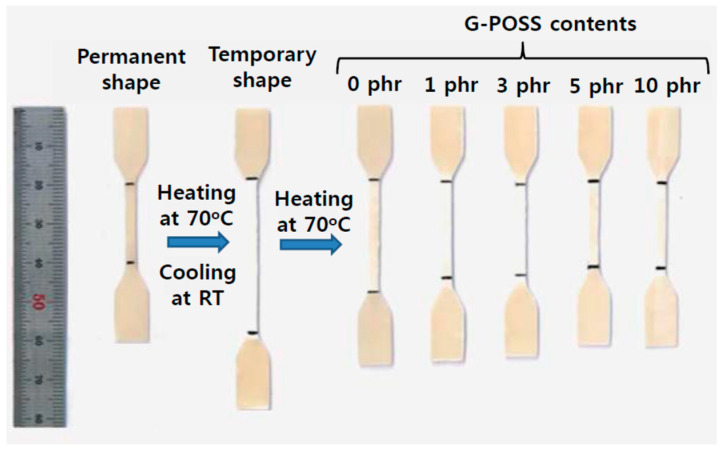
Thermally triggered shape memory procedure of samples with different POSS contents.

We observed a shape memory effect in these blends, as demonstrated in Figure 6 and in Table 3. All the temporarily elongated samples shrank and recovered to their original shape upon heating above *T*_g_ of the PA12 phase. The recovery ratio increased with increasing POSS content from 47.5% for the blend without the POSS to 94% for the blend with 10 phr POSS.

Shape memory polymers have two structural features, i.e., the cross-links that determine the permanent shape and the reversible segments acting as a switching phase [33,34]. Blend of semicrystalline PA12 with elastomer studied here have these structural features, in which PA12 crystallites with *T*_m_ of about 175 °C act as crosslink points while amorphous chain of PA12 with *T*_g_ of about 40~47 °C act as the reversible phase. When the blend was deformed under tensile load above its *T*_g_, amorphous regions are oriented and elastic energy is stored during the deformation. The stored elastic energy is released when the blends are heated above its *T*_g_, which renders the molecules to have an activity and to recover to its original shape instantaneously upon heating above the *T*_g_ and well below crystalline melting temperature. The same structural features have been reported for semicrystalline polymers like PLA [35,36] and PVDF [37] with thermally triggered shape memory effects. Amorphous portions of these semicrystalline polymer molecules can be oriented in direction to applied force at temperature higher than its *T*_g_ and much lower than its *T*_m_, and the deformed shape can be fixed as a temporary shape upon cooling below *T* < *T*_g_ due to trapping the entropy of the chains. When the temporarily fixed sample is heated above *T*_g_, the shape recovery occurs due to the release of stored entropic energy and the relaxation of the polymer molecular chains to a higher entropic state. Therefore, the driving force of shape recovery mainly stems from the elastic resilience of the elongated polymer molecular chains. This is the molecular mechanism for the inherent shape memory properties of these semicrystalline polymers. It is also to be noted that blending of these semicrystalline polymers with elastomers along with proper compatibilization can promote the orientation and reorganization of polymer chains.

The improved shape recovery of the PA12/POE blend compatibilized by POSS is correlated with the improved interfacial adhesion of PA12 with the elastomer as discussed above. The dispersed elastomer domains having good interfacial adhesion with the matrix PA12 allows the amorphous chains of the PA12 to have higher activity than those in the uncompatibilized blend and to recover to its original shape with the stress releasing instantaneously upon heating its *T*_g_.

It should be emphasized that the PA12 blend with shape memory effect and improved mechanical properties can be processed using conventional methods such as extrusion, melt spinning, injection molding and found diverse applications including heat shrinkable fibers, films, tubes as well as self-deployable and actuating devices. These materials in the form of powder are particularly suitable in metal coatings for corrosion protection as well as in powder-based 3D printing processes such as selective laser sintering (SLS) and high-speed sintering (HSS) for precise manufacturing of parts with complicated shape [38,39]. Further, this blend material can be processed with recently developed modern processing technologies, such as centrifugal spinning and pressurized gyration [40,41,42] to fabricate nonwoven shape memory webs in mass production scale for their applications in smart wearable devices, intelligent tools for biomedical applications, sensors, etc.

## 4. Conclusions

This study demonstrated that a POSS having both a glycidyl group and several alkyl groups on its cage can act as a compatibilizer for an immiscible PA12/POE (70 wt%/30 wt%) blend system. The incorporation of a small amount of G-POSS reduced the dispersed POE domain size in PA12 matrix of the blend. The combination of the decreased rubber particle size and the presence of rigid POSS nanoparticles in the blend led to interesting simultaneous improvement in modulus and toughness. Moreover, the PA12/POE blend embedded with POSS represented excellent thermally triggered shape memory effect. The PA12 blends with good mechanical properties and shape memory effects may have diverse applications such as smart packaging films, sensors, fast deployable and actuating devices. Further studies on the use of this material for modern processing technologies will be performed.

## Figures and Tables

**Table 1:** Tensile properties and Izod impact strength of samples.

G-POSS Contents (phr)	Young’s Modulus (MPa)	Tensile Strength (MPa)	Elongation at Break (%)	Toughness (MPa)	Izod Impact Strength (kJ/m^2^)
0	5 ± 0.2	18 ± 0.3	50 ± 2	853 ± 40	30 ± 0.3
1	7 ± 0.3	20 ± 0.3	66 ± 3	1216 ± 50	33 ± 0.3
3	9 ± 0.3	20 ± 0.3	68 ± 3	1245 ± 50	34 ± 0.4
5	10 ± 0.3	22 ± 0.3	71 ± 3	1323 ± 60	35 ± 0.4
10	10 ± 0.3	23 ± 0.4	63 ± 3	1365 ± 60	36 ± 0.3

**Table 2:** Glass transition temperature of samples.

G-POSS Contents (phr)	*T*_g_ of POE (°C)	*T*_g_ of PA12 (°C)
0	−53.9	47.1
1	−56.1	45.9
3	−56.3	45.8
5	−54.3	43.9
10	−56.2	41.8

**Table 3:** Shape memory properties of PA12/POE/G-POSS blend with different POSS contents.

G-POSS Contents (phr)	Shape Fixity Ratio *R*_f_ (%)	Shape Recovery Ratio *R*_r_ (%)
0	97.4	47.5
1	95.9	75.8
3	95.5	85.7
5	95.8	92.6
10	95.2	95.9

## Data Availability

The data presented in this study are available on request from the corresponding author. The data are not publicly available due to technical or time limitations at this time.
